# Metabolomics data analysis and missing value issues with application to infarcted mouse hearts

**DOI:** 10.1186/1471-2105-16-S15-P16

**Published:** 2015-10-23

**Authors:** Jasmit S Shah, Guy N Brock, Shesh N Rai

**Affiliations:** 1 The Diabetes and Obesity Center, University of Louisville, Louisville, KY 40202, USA; 2 Department of Bioinformatics and Biostatistics, University of Louisville, Louisville, KY 40202, USA

## Background

High throughput technology makes it possible to monitor metabolites on different experiments and has been widely used to detect differences in metabolites in many areas of biomedical research. Mass spectrometry has become one of the main analytical technique for profiling a wide array of compounds in the biological samples. Extracting relevant biological information from large datasets is one of the challenges. Missing values in metabolomics datasets occur widely and can arise from different sources, including both technical and biological reasons. Mostly the missing value is substituted by the minimum value, and this substitute may lead to different results in the downstream analysis. Different methods tend to give different results. In this study we summarize the statistical analysis of metabolomics data with no missing values and with missing values. With the missing values, we compare the different methods and examine the outcomes based on each method.

## Materials and methods

Analysis was done on 276 metabolites from 10 samples (12 metabolites excluded due to not detected in either group). 204 metabolites had complete information in all samples[1]. We used seven different Missing Value (MV) imputations: Zero, Mean, Median, Half Minimum (HM), k Nearest Neighbors (kNN), Random Forest (RF) and Probabilistic Principal Components Analysis (PPCA). Filtering, scaling and transformation was done with inter-quartile range, pareto scaling and log transformation respectively. Different downstream analyses such as t-test, fold change, PLS-DA, correlation analysis, etcetera, were done.

## Results

Zero gave the least number of significant metabolites whereas Mean gave the most. 55 metabolites were uniquely identified by all methods in the volcano plot; 28 metabolites were similar across all methods.

## Conclusions

We have shown that the selection of imputation methods to replace MVs may have a dramatic impact on the data. The handling of missing values is an absolutely crucial step in the data pre-processing. Metabolites such as adenylosuccinate, caprylate (8:0) and N-acetylalanine are only detectible by a specific method and may be important in their specific metabolic pathways and so choosing an appropriate method is critical. Also PLS-DA FAD is important for only kNN in predicting the class membership whereas adenine and adenylosuccinate is important only for Zero and Mean Methods. In future studies we will further examine MVs and model an appropriate method such that the correct significant metabolites are captured.
